# Hemp Seeds in Post-Arthroplasty Rehabilitation: A Pilot Clinical Study and an In Vitro Investigation

**DOI:** 10.3390/nu13124330

**Published:** 2021-11-30

**Authors:** Samantha Maurotti, Rosario Mare, Roberta Pujia, Yvelise Ferro, Elisa Mazza, Stefano Romeo, Arturo Pujia, Tiziana Montalcini

**Affiliations:** 1 Department of Health Science, University Magna Græcia, 88100 Catanzaro, Italy; smaurotti@unicz.it (S.M.); yferro@unicz.it (Y.F.); 2 Department of Medical and Surgical Science, University Magna Græcia, 88100 Catanzaro, Italy; mare@unicz.it (R.M.); roberta.puj@gmail.com (R.P.); elisamazza@unicz.it (E.M.); romeo@unicz.it (S.R.); pujia@unicz.it (A.P.); 3 Department of Molecular and Clinical Medicine, University of Gothenburg, 40530 Gothenburg, Sweden; 4 Research Center for the Prevention and Treatment of Metabolic Diseases (CR METDIS), University Magna Græcia, 88100 Catanzaro, Italy; 5 Department of Clinical and Experimental Medicine, University Magna Græcia, 88100 Catanzaro, Italy

**Keywords:** osteoarthritis, post-arthroplasty rehabilitation, bone metabolism, hemp seeds, functional food

## Abstract

Osteoarthritis is a type of degenerative joint disease that results from the breakdown of joint cartilage and underlying bone. Due to their antioxidants and anti-inflammatory action, the phytochemical constituents of many vegetable varieties could represent a new frontier for the treatment of patients with Osteoarthritis and are still being explored. The aim of this pilot human study was to investigate the effects of pasta enriched with hemp seed flour on osteoarticular pain and bone formation markers in patients in post-arthroplasty rehabilitation. Another purpose was to evaluate the effect of hemp seed extract on bone metabolism, in vitro. A pilot, controlled, clinical study was conducted to verify the feasibility of pain symptom reduction in patients with Osteoarthritis undergoing arthroplasty surgery. We also investigated the effect of hemp seed extract on the Wnt/β-catenin and ERK1/2 pathways, alkaline phosphatase, RANKL, RUNX-2, osteocalcin, and COL1A on Saos-2. After 6 weeks, the consumption of hemp seed pasta led to greater pain relief compared to the regular pasta control group (−2.9 ± 1.3 cm vs. −1.3 ± 1.3 cm; *p* = 0.02). A significant reduction in serum BALP was observed in the participants consuming the hemp seed pasta compared to control group (−2.8 ± 3.2 µg/L vs. 1.1 ± 4.3 µg/L; *p* = 0.04). In the Saos-2 cell line, hemp seed extract also upregulated Wnt/β-catenin and Erk1/2 pathways (*p* = 0.02 and *p* = 0.03) and osteoblast differentiation markers (e.g., ALP, OC, RUNX2, and COL1A) and downregulated RANKL (*p* = 0.02), compared to the control. Our study demonstrated that hemp seed can improve pain symptoms in patients with osteoarthritis undergoing arthroplasty surgery and also improves bone metabolism both in humans and in vitro. However, more clinical studies are needed to confirm our preliminary findings.

## 1. Introduction

Osteoarthritis (OA) is a painful and disabling chronic condition that represents an enormous health as well as economic burden on patients and society [1,2]. The prevalence of hip and knee OA is on the rise due to the increased aging of the population [3]. OA classically presents with symptoms of arthralgia and functional disability [4]. However, OA is a heterogeneous disease and different pathogenic pathways could play a role in the development and management of the disease [5]. It is mainly characterized by articular cartilage degradation and subchondral bone remodeling [5].

Body weight reduction and exercise are the first, non-invasive, non-pharmacological approach to managing patients with OA [6,7]. End-stage OA is treated with surgery (particularly knee and hip replacements) and weight loss is a key factor both before and after surgery in OA [8]. Unfortunately, compliance with long-term dietary changes is poor in patients affected by OA [9,10,11], and other non-invasive approaches are thus needed to meet the primary treatment goals, i.e., pain relief and limiting joint damage. Public and health professionals are increasingly interested in the importance of functional foods and nutraceuticals in diseases’ prevention. Due to their antioxidants and anti-inflammatory action, the phytochemical constituents of many fruits and vegetable varieties represent an exciting potential new frontier for the treatment of patients with OA [12]. There is some scientific evidence of the beneficial effects of specific nutritional interventions in providing symptom relief to OA patients; however, nutritional research on OA is only in its infancy and only a few ingredients have been tested to date [13]. An endocannabinoid system has been identified in OA joints, and animal studies have shown that cannabinoids reduce OA pain, inflammation, and nerve damage [14,15]. However, only a few clinical trials have tested the efficacy and safety of medical cannabis [15].

The aim of this pilot human study was, therefore, to investigate the effects of a pasta enriched with hemp seed (HS) flour on osteoarticular pain and bone formation markers in patients with OA in post-arthroplasty rehabilitation. An additional aim was to evaluate the effect of an HS extract (HSE) in vitro on bone metabolism. 

## 2. Materials and Methods

### 2.1. Human Study Design

We conducted a pilot intervention study by enrolling 18 white volunteers of both genders, aged 50–85 years, in a post-hospital rehabilitation period due to total hip (THR) or total knee replacements (TKR). They were recruited through a local advertisement between June and October 2019. A sample of seven consecutive patients attending the “FKTSalus, srl” Rehabilitation and Physiotherapy Center in Crotone, Italy, was enrolled to receive 100 g per day of a HS whole-grain pasta (provided by Astorino Pasta, srl, Crotone, Italy) for 6 weeks (enrolment period was between 11 July and 8 October 2019). A sample of 11 individuals of usual diet and not taking HS pasta served as control (regular pasta control group). Written, informed consent was obtained from all participants included in the study. The protocol was approved by the local ethical committee at the “Mater Domini” Azienda University Hospital (05/2018/CE, approved 18 January 2018). The investigation conformed to the principles outlined in the declaration of Helsinki. Subjects were excluded if they presented any clinical condition affecting bone metabolism (such as kidney, liver, thyroid or parathyroids, malabsorption syndromes, rheumatic diseases, malignant tumors, or hematological diseases). No patients were taking dietary supplements, anti-osteoporotic agents, psychotropic drugs, glucocorticoids, estrogens, aromatase inhibitors, thyroid hormone, antiepileptics, fluoride, or calcitonin as ascertained from the visit and their medical records. If participants took calcium and/or vitamin D supplementation at the time of enrolment, they continued on the same dose throughout the duration of the study. 

In this study, we used a Visual Analog Scale (VAS) to measure pain, which consisted of a self-reported pain-rating scale. Specifically, a single, handwritten mark was placed by participants at one point along the length of a 10-cm line that represented a continuum between the two ends of the scale (“no pain” on the left end at 0 cm of the scale and the “worst pain” on the right end of the scale at 10 cm) [16].

Primary outcomes of the study were change in VAS and in key bone turnover markers such as serum BALP and osteocalcin.

Body mass index (BMI, kg/m^2^; dry weight in meters squared), systemic blood pressure, and the presence of any risk factors for bone loss, such as diabetes and smoking habit, were assessed from medical records.

#### 2.1.1. Raw Material for Pasta Manufacturing and Protein Content Analysis

HS pasta was produced by Astorino Pasta srl (Crotone, Italy). HS pasta was manufactured with 85% whole durum wheat (Triticum durum) semolina and 15% HS flour. HS pasta was produced following the standard pasta-making process steps, i.e., hydration, mixing (25/30 min), and extrusion (60 °C) in a press (Pama Roma, Roma, Italy) and drying at low temperature (40/42 °C, 16/18 h) in a drier (La Parmigiana srl, Parma, Italy). Optimal cooking time for HS whole-grain pasta was 12–15 min. Proteins’ content in the HS pasta, which was assessed by high-performance liquid chromatography (UltiMate 3000 Standard HPLC System, Thermo Fisher Scientific, Italy), was 15.17%/100 g.

#### 2.1.2. Biochemical Assessments

Venous blood was collected after fasting overnight into vacutainer tubes (Becton & Dickinson, Plymouth, England) and centrifuged within 4 h. Serum glucose, total cholesterol (TC), high-density lipoprotein cholesterol (HDL-C), triglycerides, creatinine, and transaminases were measured by chemiluminescent immunoassay on COBAS 8000 (Roche, Switzerland), at baseline and after 6 weeks, according to the manufacturer’s instructions. We used fasting lipid levels to calculate the value for LDL-C (Friedewald formula). 

BALP and osteocalcin were performed by chemiluminescent immunoassay on Liaison^®^ XL (DiaSorin, Italy), at baseline and after 6 weeks, according to the manufacturer’s instructions.

Interleukin-1β (IL-1β) and interleukin-10 (IL-10) were assessed by ELISA Kit (R&D Systems, Minneapolis, MN, USA). Quality control was assessed daily for all determinations.

#### 2.1.3. Bioavailability Assessment

Prior to the clinical intervention study, we performed a bioavailability study at the Nutrition Unit of the “Mater Domini” University Hospital. We assessed the bioavailability of HS vegetable proteins in humans by HPLC. We enrolled a sample of three healthy volunteers who were not affected by malabsorption syndrome, and, in the case of the female (n = 1 female), not pregnant. The average age was 33 ± 8 years and BMI was 23.6 ± 2 kg/m^2^. After a basal blood sampling, each subject was given a 100 g per day of HS pasta for 3 consecutive days. All subjects signed an informed consent to participate at this step of the study.

### 2.2. In Vitro Study

#### 2.2.1. Chemicals, Reagents, and Materials

Hemp seeds were bought in local store, “Mondo Sativa”, Catanzaro, Italy. Dexamethasone (DEX), bicinchoninic acid assay kit (BCA), and Bradford reagent for protein quantification were purchased by Sigma-Aldrich (St. Louis MO, USA) as well as Folin–Ciocalteu reagent, phenol, sodium carbonate, gallic acid, and 2,2-diphenyl-1-picrylhydrazyl (DPPH) required for spectrophotometric assays. McCoy’s 5A modified medium, fetal bovine serum (FBS), and trypsin-EDTA were purchased from Gibco (Life technologies, Grand Island, NY, USA); penicillin/streptomycin was purchased from Lonza (Basel, Switzerland). Methanol and all other solvents and reagents were of analytical grade (Carlo Erba, Milan, Italy).

#### 2.2.2. Hemp Seeds’ Extraction

Hemp (Cannabis sativa L.) seeds’ extract (HSE) was obtained at room temperature as previously described by Wang and coworkers [17]. Briefly, HS (100 grams) were mixed with 1.5 L milliQ^®^ water and pH was adjusted to 10 with 1 N sodium hydroxide solution. The suspension obtained was maintained under continuous stirring for 60 min and subsequently centrifuged at 8000× *g* for 30 min. The pellet was discarded while supernatant’s pH was adjusted to pH 5 with 1N hydrochloric acid solution and temperature was kept at 4 °C, in order to obtain a protein-rich precipitate. Precipitates were collected by centrifugation process (6500× *g*, 25 min), washed with pre-cooled deionized water, and finally re-dispersed in the same medium. All dispersions were treated with suitable solutions, in order to obtain a final pH of 7 and subsequently quickly frozen (−80 °C). The freeze-drying technique was used in order to obtained dried extracts with residue water content less than 4%. The freeze dryer apparatus was a SpeedVac-Vacuum Concentrator (Thermo Scientific™). HSE were finally collected and stored in a freezer (−20 °C).

#### 2.2.3. Determination of Proteins, Carbohydrates, and Total Phenolic Content

Proteins contained in HSE were quantified by two different methods including Bicinchoninic Acid Protein Assay (BCA proteins assay kit, Sigma-Aldrich) and Bradford Protein Assay. The BCA protein assay was performed as described in literature [18]. Bradford assay for protein determination was performed with slight modifications to the original assay [19]. Carbohydrates contained in HSE were quantified by the Phenol-Sulfuric Acid method as previously performed by Albalasmeh and coworkers, with slight differences [20]. Total Phenolic Content (TPC) in HSE (including hydrolysable tannins and polyphenols) was quantified as previously proposed by Aruwa and his collaborators [21]. The antioxidant activity of HSE, quantified by DPPH assay, aimed to investigate the capability of our extracts to inhibit free radicals. DPPH solution and L-ascorbic acid solution (5 mg/mL) were used, respectively, as negative and positive controls. The percentage radical scavenging activity was normalized as a function of HSE concentrations. The percentage DPPH inhibition was calculated using the following equation [22]:

I(%) = [(A0–A1)/A0] × 100

A0 = Absorbance of negative control A1 = Absorbance of extracts/standards.

All quantifications were performed with a spectrophotometer (UV-Vis Genesys 150^®^-ThermoScientific).

#### 2.2.4. Cell Line and Culture Conditions

In the present study, Saos-2 “human osteoblast-like” cell line was obtained from ATCC. The cells were maintained in McCoy’s 5A (Gibco, Carlsbad, CA, USA) supplemented with 15% fetal bovine serum (Gibco, Carlsbad, CA, USA) and 1% penicillin streptomycin (PAA, Linz, Austria), at 37 °C in 5% CO_2_, then harvested by trypsinization and subcultured twice weekly. In all the experiments Saos-2 cells were incubated with dexamethasone 10 nM (Sigma Aldrich, St. Louis, MO, USA) to obtain a more differentiated cell line.

#### 2.2.5. Cell Viability Assay

To evaluate cell viability, Saos-2 cells were seeded at a density of 1 × 10^4^ cells/well in 96-well plates. Cells were grown in serum-free medium and incubated with HSE at concentration of 5, 10, and 20 µg/mL for 24 h. Cell viability was determined by 3-(4,5-dimethylthiazol-2- yl)-2,5-diphenyltetrazolium bromide (MTT) assay. Briefly, MTT (Sigma, St. Louis, MO, USA) solution (5 mg/mL) was added to each well and incubated at 37 °C for 4 h. The supernatants were then removed and replaced by 100 μL of DMSO. The optical density (OD) was measured at a wavelength of 570 nm.

#### 2.2.6. Western Blotting

Saos-2 cells were seeded at a density of 2 × 10^5^ cells/well in six-well dishes and 1 × 10^6^ cells/well in 100-mm culture dishes. Cells were grown in serum-free medium and incubated with HSE 5, 10, or 20 µg/mL for 10 min or 24 h. Cells were lysed in Mammalian Protein Extraction Reagent (M-PER) (Pierce, Thermo Fisher Scientific). Medium from cell cultures was centrifuged by using Amicon Ultra Centrifugal Filters (EMD Millipore, USA) to concentrate the extracellular proteins of the cell medium. Western blot analysis of proteins from cell lysates and from cell medium was performed according to standard procedures. The following antibodies were used: rabbit anti β-catenin (19,807), rabbit anti-p Extracellular Signal-regulated Kinase (ERK)1/2 (9101), and mouse anti- β-actin (3700), by Cell Signaling Technology (Beverly, MA, USA); rabbit anti-type I collagen (COL1) (HPA011795) and mouse anti-Albumin (A6684), from Sigma Aldrich (St. Louis, MO, USA); and mouse anti-Alkaline phosphatase (ALP), by Abcam (Cambridge).

#### 2.2.7. Alkaline Phosphatase (ALP) Activity

Saos-2 cells were seeded at a density of 2 × 10^5^ cells/well in six-well dishes. Cells were grown in serum-free medium with HSE at concentrations of 5, 10, or 20 µg/mL for 24 h and dexamethasone 10 nM for 24 hours. Cells were lysed in M-PER Reagent for protein extraction (Pierce, Thermo Fisher Scientific). The lysates were centrifuged at 14,000× *g* for 8 min at +4 °C. After centrifugation at 12,000× *g* at 4 °C for 10 min, the supernatant was collected and the pollution was evaluated by the Bradford assay (BioRad,) and alkaline phosphatase (ALP) activity was determined by the p-nitrophenyl phosphate (pNPP) colorimetric method (cat.KA0817, Abnova, Walnut, CA, USA).

#### 2.2.8. Real Time-PCR

Saos-2 cells were seeded at a density of 1 × 10^6^ cells/well in 100-mm culture dishes. Cells were grown in serum-free medium with HSE at concentrations of 5, 10, or 20 µg/mL for 24 h and dexamethasone 10 nM for 24 hours. Total RNA from cells was extracted with Trizol reagent (Life technologies, UK) according to manufacturer’s instructions. The cDNA was synthesized from 1 µg of total RNA, using High-Capacity cDNA Reverse Transcription Kit (Applied Biosystems, Foster City, CA, USA). The mRNA expression of Receptor activator of nuclear factor kappa-Β ligand (RANKL), osteoprotegerin (OPG), Runt-related transcription factor 2 (RUNX2), Osteocalcin (OC), and β-ACTIN was quantified by real time-PCR using SYBR^®^ Green dye (SYBR^®^ Green PCR Master Mix, Applied Biosystems, Foster City, CA, USA) (Table S1).

### 2.3. Statistical Analysis

Data are reported as mean ± standard deviation (SD). To find at least 20% (~3 µg/L) [23] difference in serum BALP between the groups, considering a SD of 3 µg/L [23], with an effect size (ES) of 1, with 80% power on a two-sided level of significance, 10 participants for each group are required.

For the human study, a Chi square test was performed to analyse the prevalence between groups and an unpaired Student’s *t*-test was used to compare the difference between means. Changes in the clinical characteristics at baseline and follow-up (between group variation) were calculated using unpaired Student’s *t*-test (two tailed). Specifically, we calculated the changes in variables (such as VAS, BALP, osteocalcin, IL-1β, and IL-10) and compared the means of these changes between treatment groups. Normal distribution of the variables was tested using the Kolmogorov–Smirnov test. Nonparametric Mann–Whitney test was used to evaluate the differences of IL-1β and IL-10 changes between groups. We used a patient interview to assess adherence to the treatment. All comparisons were performed using SPSS 25.0 for Windows (IBM Corporation, New York, NY, USA).

For the in vitro study, data resulted from a mean of at least three independent experiments and were analyzed with GraphPad Prism 5.0 software using a two-tailed Student’s t test and linear regression test.

In both the studies, significant differences were assumed to be present at *p* <  0.05 (two-tailed).

## 3. Results

### 3.1. Bioavailability of Proteins after HS Pasta Consumption

The bioavailability analysis of HS vegetable proteins found their presence in the serum (5.4 ± 0.9 µg/mL, increase from baseline). All subjects took the HS pasta prototype as indicated and did not develop any adverse effects after taking it.

### 3.2. Clinical Characteristics of Participants in the Pilot Study

The mean age of the enrolled population was 72 ± 8 years. The mean basal VAS was 6.8 ± 2 cm, BALP was 11.7 ± 4 µg/L, and osteocalcin was 20.9 ± 11 ng/mL. All enrolled women were in menopause, and 56% of the participants had undergone THR. Participants’ demographic and clinical characteristics according to treatment are shown in Table 1. The groups were comparable for all characteristics including prevalence of nonsteroidal anti-inflammatory drugs’ (NSAIDs) use (Table 1).

### 3.3. Clinical Characteristics Changes at Follow-Up and Outcomes of the Study

**Figure 1:**
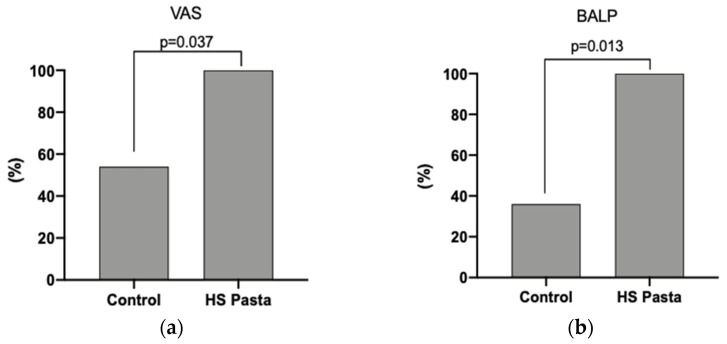
Prevalence of subjects who had a reduction in VAS value (**a**) and serum BALP concentration (**b**) after 6 weeks of treatment. A total of 18 participants were enrolled (control group n = 11, HS pasta n = 7, respectively). Abbreviation: VAS = visual analog scale; BALP = bone-specific alkaline phosphatase.

Changes in the clinical parameters after each treatment period are shown in Table 2. After 6 weeks, the consumption of HS pasta resulted in a greater VAS change compared to the regular pasta control group (−2.9 ± 1.3 cm and−1.3 ± 1.3 cm, *p* = 0.028, respectively, Table 2). Furthermore, all subjects who consumed the HS pasta had a significant reduction in VAS value in comparison with 54% of the control group (*p* = 0.037; Figure 1a). A significant reduction of serum BALP concentration was also detected in the participants consuming the HS pasta compared to individuals consuming their regular pasta (*p* = 0.041; Table 2). In particular, BALP change was −2.8 ± 3.2 µg/L and 1.1 ± 4.3 µg/L in the HS pasta and in the regular pasta control group, respectively (Table 2). Moreover, we found that 100% of patients who ate HS pasta had a reduction in serum BALP concentration compared to 36% of patients in the regular pasta control group (*p* = 0.013, Figure 1b).

Prevalence of NSAIDs was reduced in both groups at the follow-up (29% and 36% in the HS pasta and in the regular pasta control group, *p* = 1, respectively). No other variables were significantly different between groups after 6 weeks of treatment (Table 2). The participants in the HS pasta group had a high adherence to the protocol (i.e., >80% of the prescribed treatment) and did not develop any adverse effects during 6 weeks of treatment.

### 3.4. Characterization of Hemp Seeds’ Extract

HSE was characterized in term of macronutrients that are shown in Table 3. In particular, proteins represented 15.56% w/w of the extract according to Bradford assay, a trend confirmed by BCA assay, which resulted in 14.11% w/w of proteins into the extract (Table 3). Carbohydrates, determined by Phenol-Sulfuric Acid method, represented 21.2% w/w of the extract, while Folin–Ciocalteu reagent revealed the presence of polyphenols for 29.42% w/w with a significant inhibiting power of free radicals (37.02% compared to the positive control). The exceeding portion, indicated in Table 3 as “other”, should represent lipids and wastes deriving from the extractive procedures such as fatty acids obtained from the plausible hydrolysis of cell membrane phospholipids, which were not separable from other macromolecules or quantifiable by commonly used spectrophotometric techniques.

### 3.5. Hemp Seeds’ Extract Does Not Act on Osteoblasts’ Proliferation In Vitro

To test the hypothesis that HSE increases osteoblasts’ proliferation, cells were incubated with HSE at 5, 10, or 20 µg/mL for 24 h and cell proliferation was measured by 3-(4,5-dimethylthiazol-2-yl)-2,5-diphenyltetrazolium bromide (MTT) assay. Cells treated with HSE did not affect cell proliferation at 24 h in comparison to the control (Figure S1).

### 3.6. Hemp Seeds’ Extract Induces Higher β-Catenin and p-ERK1/2 Levels in Saos-2

**Figure 2:**
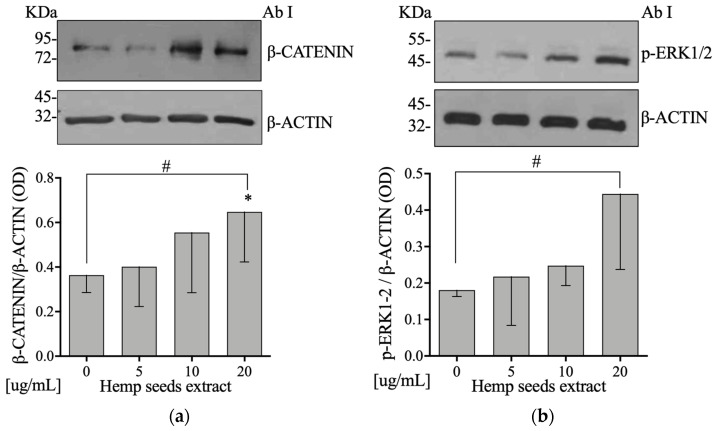
Hemp seed extract increases β-catenin and p-ERK1/2 pathways protein levels on Saos-2 cells. (**a**) Semi-confluent cultures of human osteoblast-like cells (Saos-2) incubated with HSE 5, 10, or 20 µg/mL for 24 hours. Cell proteins were analyzed by Western blotting with antibodies specific to β-catenin and β-actin. (**b**) Semi-confluent cultures of human osteoblast-like cells (Saos-2) were incubated with HSE 5, 10, or 20 µg/mL for 10 min. Cell proteins were analyzed by Western blotting with antibodies specific to phosphorylated ERK1/2 and β-actin. Data are represented as mean ± SD. Statistical analysis: Student’s t-test vs. 0 * *p* < 0.05, Linear regression # *p* < 0.05.

To test the hypothesis that HSE activates β-catenin and p-ERK1/2 pathways, Saos-2 cells were incubated with HSE at 5, 10, or 20 µg/mL for 24 h and 10 min, respectively. HSE incubation increased the protein expression levels of β-catenin and p-ERK1/2 in a dose-dependent manner in comparison to the control (linear regression: *p* = 0.036 and *p* = 0.028, respectively) (Figure 2a,b).

### 3.7. Hemp Seeds’ Extract Increases Activity and Protein Levels of Alkaline Phosphatase in Saos-2

**Figure 3:**
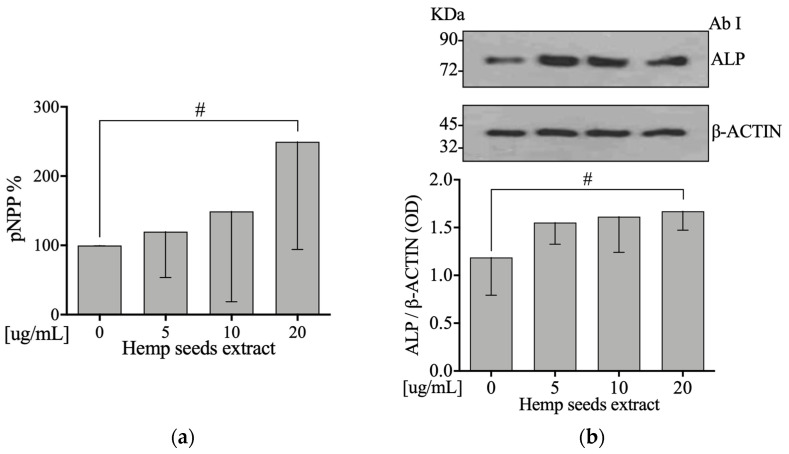
HSE increases activity and protein levels of Alkaline Phosphatase on Saos-2 cells. Semi-confluent cultures of human osteoblast-like cells (Saos-2) were incubated with HSE 5, 10, or 20 µg/mL for 24 hours. (**a**) ALP activity was measured by pNPP method. (**b**) Cell proteins were analyzed by Western blotting with antibodies specific to alkaline phosphatase and β-actin. Data are represented as mean ± SD. Statistical analysis: Linear regression # *p* < 0.05.

To test the hypothesis that HSE affects osteoblasts’ differentiation, protein expression levels of ALP were measured. Moreover, ALP activity was determined on cell lysate by using p-Nitrophenyl Phosphate (pNPP). After incubation with HSE at 5, 10, or 20 µg/mL for 24 h, we did observe, in Saos-2 cells, an increase on ALP activity and protein expression levels in a dose-dependent manner in comparison to the control (Linear Regression: *p* = 0.039 and *p* = 0.026, respectively) (Figure 3a,b).

### 3.8. Hemp Seeds’ Extract Decreases RANKL and Increases RUNX2 and Osteocalcin mRNA Expression Levels in Saos-2

**Figure 4:**
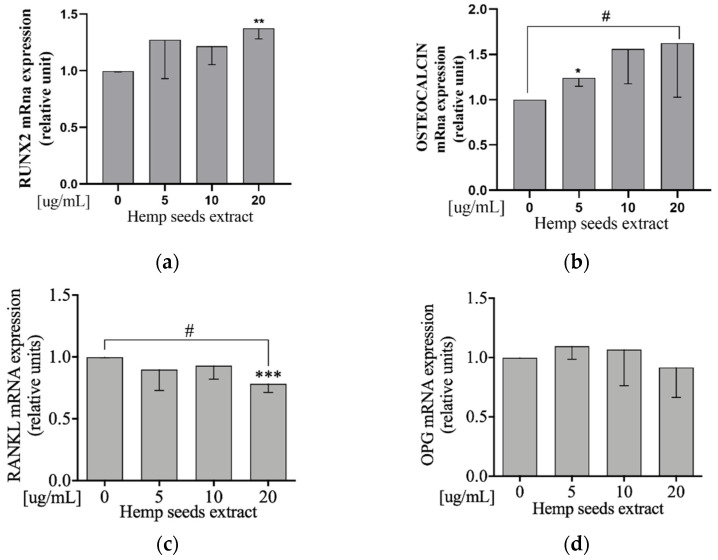
HSE downregulates RANKL and upregulates RUNX2 and Osteocalcin mRna expression levels on Saos-2 cells. Semi-confluent cultures of human osteoblast-like cells (Saos-2) were incubated with HSE 5, 10, or 20 µg/mL for 24 h. The mRNA expression levels of RANKL (**c**), OPG (**d**), RUNX2 (**a**), and Osteocalcin (**b**) were measured by RT-PCR. Data were analyzed using the 2-ΔΔCq method and normalized to β-actin. Data are represented as mean ± SD. Statistical analysis: Student’s *t*-test vs. 0 * *p* < 0.05; ** *p* < 0.01; *** *p* < 0.001, Linear regression # *p* < 0.05.

Saos2 cells were exposed to HSE at 5, 10, or 20 µg/mL doses. Only the 20-µg/mL dose decreased RANKL and increased RUNX2 mRna levels, compared to the control (Student’s *t*-test: *p* = 0.0008 and *p* = 0.002, respectively) (Figure 4a,c). Furthermore, we did observe a decreased RANKL and increased Osteocalcin mRNA expression level in a dose-dependent manner in comparison to the control (Linear Regression: *p* = 0.023 and *p* = 0.044, respectively; Figure 4b,c). HSE did not affect OPG mRNA expression (Figure 4d).

### 3.9. Hemp Seeds’ Extract Increases Extracellular and Intracellular COL1A Protein Levels in Saos-2

**Figure 5:**
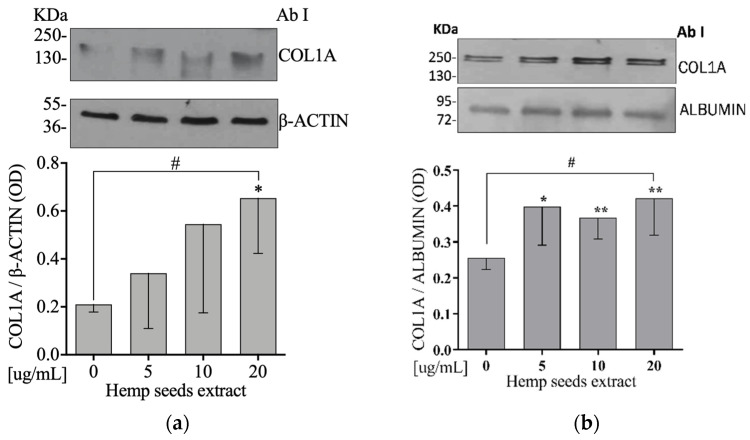
HSE increases intra- and extracellular COL1A protein levels on Saos-2 cells. Semi-confluent cultures of human osteoblast-like cells (Saos-2) were incubated with HSE 5, 10, or 20 µg/mL for 24 h. Cell and medium proteins ((**a**,**b**), respectively) were analyzed by Western blotting, with antibodies specific to COL1A, β-actin, and Albumin. Data are represented as mean ± SD. Statistical analysis: Student’s *t*-test vs. 0 * *p* < 0.05 ** *p* < 0.01, Linear regression # *p* < 0.05.

To test the hypothesis that HSE acts on bone matrix, Saos-2 cells were exposed to 5, 10, or 20 µg/mL of HSE for 24 h. We did observe an increase on both intra- and extracellular COL1A protein expression levels (Figure 5a,b, respectively) in a dose-dependent manner in comparison to the control (linear regression: *p* = 0.043 and *p* = 0.010, respectively).

## 4. Discussion

This pilot study was conducted on humans and investigated, for the first time, the feasibility of treating patients with OA undergoing arthroprosthesis with a functional food. The intervention consisted of an HS pasta containing HS flour, which, compared to regular pasta, reduced the painful manifestations in these patients in post-surgery rehabilitation (Table 2). In addition, HS pasta improved the bone turnover involved in the joint, as demonstrated by the BALP reduction (Table 2). Since the effects of HS on OA have never been studied in humans, our results contribute to providing new scientific evidence on this topic.

Participants in the HS pasta group had a high protocol adherence (i.e., >80% of the prescribed treatment) and developed no adverse effects during the 6 weeks of treatment.

BALP is an important biomarker of bone formation produced by osteoblasts, which appears to be higher in individuals with OA [24], and may be associated with chronic pain [25]. In fact, the painful symptoms are caused not only by the degradation of the cartilage, but also by excessive resorption of the subchondral bone and BALP increases [26,27,28]. 

The effects on the perception of pain and on the reduction of BALP may be partly attributable to the action of cannabinoids present in HS [29,30]. In fact, the endocannabinoid system (ECS) in bone metabolism appears to play a key role [31] in modulating the pathophysiology of OA and in reducing painful symptoms related to OA [15]. The ECS is, therefore, considered as a possible therapeutic target for pain management. However, the concentration of cannabinoids was not assessed in the study. 

Bioactive substances such as polyphenols and phenolic compounds, proteins, and minerals, also present in HS [32], can contribute to improving painful symptoms in patients with OA, improving their quality of life [33,34,35]. These compounds act rapidly, even only after 28 days of treatment [36]. Another recent study showed a rapid reduction in BALP after the intake of a functional food rich in antioxidant molecules [23]. 

Due to the changes in BALP induced by the HS pasta and in the light of previous studies showing excessive resorption of subchondral bone in OA, which also promotes disease progression [37,38], we evaluated the effects of the HSE on osteoblast metabolism.

We first characterized the HSE, which appeared to contain 16% of proteins, 21% of carbohydrates, and 29% of polyphenols (total content), with a significant inhibitory power of free radicals (37% compared to the positive control) (Table 3).

The results of the HSE characterization were in line with the literature data on the high nutritional value of HS and the presence of numerous bioactive molecules [32,39,40].

In our in vitro study, we evaluated the effects of HSE on the Wnt/β-catenin pathway, which represents an important pathway in the bone formation, playing a central role in the regulation of osteoblast differentiation [41]. The Wnt/β-catenin pathway also mediates the up-regulation of osteoprotegerin and the down-regulation of RANKL in osteoblast cells, leading to the inhibition of bone resorption [41], and it also plays a role in controlling the osteoblast differentiation via RUNX2 expression [42,43].

In line with the literature, our study showed that HSE increased the expression of Wnt/β-catenin protein (Figure 2a) and RUNX2 gene, also reducing RANKL gene expression (Figure 4c).

These results may be explained by the presence of phenolic compounds and polyphenols in the HSE [32], which facilitates the accumulation of Wnt/β-catenin in the cytosol and its nuclear translocation, favoring bone formation [44]. In line with our results, other studies have demonstrated that polyphenols inhibit bone resorption by reducing RANKL expression [44] and phenolic compounds, such as caffeic acid and p-hydroxycinnamic acid, and influence RANKL expression, respectively decreasing RANKL/OPG signal [45] and suppressing osteoclastogenesis by the antagonization of RANKL-induced NF-κB activation [46]. Furthermore, protocatechuic acid and caffeic acid upregulate β-catenin-induced RUNX2 [45,47]. In addition, in vivo, cannabidiol treatment also increases β-catenin and reduces RANKL [48].

Another result that emerged from our in vitro study was that HSE is able to increase the levels of p-ERK1/2 protein expression (Figure 2b), a signaling pathway that is known to be involved in the differentiation of osteoblasts [49]. This effect may be attributable to the presence of acid glutamic [40,50] and phenolic compounds in HS through their ROS scavenger activity [51,52,53,54,55,56]. In support of our hypothesis, Xiao et al. demonstrated that vanillic acid, a phenolic compound, upregulates RUNX2, osteocalcin, and OPG through phosphorylation of ERK1/2 [57].

In addition, phenolic compounds stimulate the differentiation, mineralization, and activity of ALP [58].

In the light of these results, the effect of HS in modulating these signaling pathways is an important finding, which could have therapeutic applications.

Another result obtained after treatment of osteoblasts with HSE was the increase in ALP (Figure 3a,b) as well as the intra- and extracellular COL1A (Figure 5a,b). ALP is involved in the formation of the bone matrix, which is one of the most important biomarkers of osteoblast activity and maturation [59]. COL1A is the main component of the bone matrix, which helps to counteract the deformation and fracture mechanisms of the bone [60]. Torricelli et al. showed that arginine, which is present in HS [40], increases several markers of bone formation including ALP and COL1A [61]. Precisely, glutamic acid, which is involved in the biosynthesis of arginine, acts on the osteogenic differentiation of mesenchymal stem cells (MSC) by increasing the expression of ALP [62].

Several studies confirm that HS, with its mix of bioactive molecules, promotes the synthesis of the bone matrix protein and the differentiation of osteoblasts [63,64,65]. This effect could be related to phenolic compounds, as ferulic acid, which was already demonstrated to increase ALP activity and synthesis of Type I collagen [66]. 

However, our study has some limitations. HSE was characterized in terms of carbohydrates, proteins, and polyphenols, but we did not analyze the concentration of the individual amino acids or polyphenols and neither did we measure the concentration of cannabinoids present in HS. 

In relation to the pilot study on humans, the number of subjects enrolled was limited precisely because it is a feasibility study, but sufficient to generate new hypotheses. The results are, thus, preliminary and cannot be applied to other categories such as premenopausal women or subjects with osteoporosis. In addition, only some bone markers, and not markers of bone resorption and oxidative stress, were evaluated in this study. In fact, it was demonstrated that activation of bone resorption is evident in the subchondral bone microenvironment in early-stage OA, while late-stage OA, as in our participants, is characterized by a bias towards activation of bone formation activity [67]. As a consequence, we chose to perform our experiment only on human osteoblast. For the same reasons, we did not assess bone resorption serum markers.

We mainly evaluated the feasibility of an innovative therapeutic approach; thus, the results should be interpreted with caution. The presence of other dietary factors and components that could have influenced the results cannot be excluded.

Despite these limitations, we believe that the results obtained in this study are very encouraging and original and generate new hypotheses for further in vitro studies and ad hoc studies on humans.

## 5. Conclusions

Our study demonstrates, for the first time, that HS can improve pain symptoms and markers of bone metabolism in patients with OA undergoing arthroplasty surgery. The observed results, both in vitro and in humans, may be due to the high nutritional value of HS, as well as to the presence of various bioactive compounds. In conclusion, a functional food with HS could have positive effects on the osteo-articular system without causing adverse events. However, more clinical studies are needed to confirm our findings.

## Figures and Tables

**Table 1:** Baseline demographic and clinical characteristics of participants according to the treatments.

Variables	Control (n = 11)	HS Pasta (n = 7)	*p*-Value
Age (year)	75 ± 7	69 ± 10	0.21
VAS (cm)	6.6 ± 2	7.1 ± 2	0.64
Weight (Kg)	80.1 ± 14	78.3 ± 14	0.79
BMI (Kg/m^2^)	31.2 ± 5	28.3 ± 3	0.17
HG (Kg)	23.0 ± 7	27.2 ± 16	0.52
SBP (mmHg)	130 ± 11	135 ± 13	0.47
DBP (mmHg)	82 ± 11	78 ± 7	0.38
Biochemical evaluation			
Glucose (mg/dL)	109 ± 22	113 ± 25	0.73
Creatinine (mg/dL)	0.83 ± 0.2	0.85 ± 0.1	0.72
TC (mg/dL)	173 ± 38	174 ± 31	0.93
HDL-C (mg/dL)	45 ± 14	47±7	0.75
LDL-C (mg/dL)	104 ± 35	100 ± 31	0.77
TG (mg/dL)	117 ± 49	138 ± 72	0.49
AST (IU/L)	22 ± 12	18 ± 5	0.43
ALT (IU/L)	15 ± 11	15 ± 5	0.86
Osteocalcin (ng/mL)	21.7 ± 13	19.8 ± 10	0.73
BALP (µg/L)	10.8 ± 4	13.2 ± 5	0.29
Lymphocyte (×10^3^/µL)	1.7 ± 0.7	2.2 ± 0.7	0.16
Monocyte (×10^3^/µL)	0.38 ± 0.1	0.39 ± 0.1	0.89
Cytokine evaluation			
IL-1β (pg/mL)	2.3 ± 0.5	2.2 ± 0.4	0.86
IL-10 (pg/mL)	0.69 ± 0.3	0.71 ± 0.2	0.47
Prevalence			
Gender, Male (%)	36	71	0.35
Smokers (%)	9	0	1
THR (%)	36	86	0.06
Hyperlipidemia (%)	45	57	1
Hypertension (%)	73	57	0.62
T2D (%)	36	29	1
Medications			
NSAIDs (%)	91	86	1
Calcium (%)	0	14	0.38
Vitamin D (%)	18	14	1

Abbreviations: VAS = visual analog scale; BMI = body mass index; HG = handgrip strength; SBP = Systolic Blood Pressure; DBP = Diastolic Blood Pressure; TC = total cholesterol; TG = triglycerides; HDL-C = high-density lipoprotein cholesterol; LDL-C = low-density lipoprotein cholesterol; AST = aspartate aminotransferase; ALT = alanine aminotransferase; BALP = bone-specific alkaline phosphatase; IL-1β = interleukin-1β; IL-10 = interleukin-10; THR = total hip replacements; T2D = type 2 diabetes; NSAIDs = nonsteroidal anti-inflammatory drugs.

**Table 2:** Changes of the characteristics of participants at follow-up according to intervention.

Variables	Control (n = 11)	HS Pasta (n = 7)	*p*-Value
VAS (cm)	−1.3 ± 1.3	−2.9 ± 1.3	0.028
Weight (Kg)	0.4 ± 1.7	−0.9 ± 1.9	0.17
BMI (Kg/m^2^)	0.1 ± 0.7	−0.3 ± 0.9	0.32
HG (Kg)	0.5 ± 1.8	0.2 ± 0.4	0.61
SBP (mmHg)	1 ± 9	−2 ± 4	0.38
DBP (mmHg)	4 ± 5	3 ± 5	0.85
Biochemical evaluation			
Glucose (mg/dL)	8 ± 28	0 ± 5	0.38
Creatinine (mg/dL)	−0.01 ± 0.1	0.01 ± 0.1	0.64
TC (mg/dL)	9 ± 23	1 ± 21	0.42
HDL-C (mg/dL)	2 ± 5	1 ± 1	0.30
LDL-C (mg/dL)	5 ± 19	−1 ± 18	0.55
TG (mg/dL)	12 ± 23	7 ± 28	0.73
AST (IU/L)	−1 ± 4	−0.7 ± 2	0.75
ALT (IU/L)	−1 ± 4	1 ± 2	0.09
Osteocalcin (ng/mL)	−1.2 ± 8.5	2.6 ± 8.4	0.38
BALP (µg/L)	1.1 ± 4.3	−2.8 ± 3.2	0.041
Lymphocyte (×10ˆ3/µL)	−0.03 ± 0.2	0.01 ± 0.2	0.71
Monocyte (×10ˆ3/µL)	−0.05 ± 0.1	−0.01 ± 0.1	0.40
Cytokine evaluation			
IL-1β (pg/mL)	0.3 ± 0.2	0.5 ± 0.3	0.12
IL-10 (pg/mL)	−0.001 ± 0.2	0.001 ± 0.2	0.65

Abbreviation: VAS = visual analog scale; BMI = body mass index; HG = handgrip strength; SBP = Systolic Blood Pressure; DBP = Diastolic Blood Pressure; TC = total cholesterol; TG = triglycerides; HDL-C = high-density lipoprotein cholesterol; LDL-C = low-density lipoprotein cholesterol; AST = aspartate aminotransferase; ALT = alanine aminotransferase; BALP = bone-specific alkaline phosphatase; IL-1β = interleukin-1β; IL-10 = interleukin-10.

**Table 3:** Chemical composition of Hemp seeds’ extract (% w/w).

Molecules	Mean ± SD	% (w/w)
Proteins (μg/mL)	5.65 ± 0.01 * 4.67 ± 0.002 δ	14.1 15.6
Carbohydrates (μg)	169.35 ± 2.41	21.2
Polyphenols (GAE-μg)	0.0735 ± 0.0008	29.4
Others	-	~33.8
Antioxidant activity (I%)	-	37.0

Note. Proteins contained were quantified by * Bicinchoninic Acid Protein assay and ^δ^ Bradford proteins assay. Abbreviation: I% = percentage of inhibition.

## Data Availability

The data presented in this study are available on request from the corresponding author.

## Supplementary Materials

The following are available online at https://www.mdpi.com/article/10.3390/nu13124330/s1, Table S1: Real-Time primer sequences. Figure S1: HSE does not increase cell proliferation of Saos-2 cells.
