# Supplementary material for: Hemp Seeds in Post-Arthroplasty Rehabilitation: A Pilot Clinical Study and an In Vitro Investigation
Source: Nutrients. 2021 Nov 30;13(12):4330. doi: 10.3390/nu13124330 (PMC8709006; doi:10.3390/nu13124330)
Supplement: Supplementary file 1 [file nutrients-13-04330-s001.zip › nutrients-1432794-SI.pdf]

Supplemental Table S1. *Real-Time primer sequences*

Gene	Forward	Reverse
RANKL	5'-AGAGCGCAGATGGATCCTAA-3'	5'-TTCCTTTTGCACAGCTCCTT-3'
Osteoprotegerin	5'-TGCAGTACGTCAAGCAGGAG-3'	5'-GTGTCTTGGTCGCCATTTTT-3'
RUNX2	5'-TTACTTACACCCCGCCAGTC-3'	5'-TATGGAGTGCTGCTGGTCTG-3'
Osteocalcin	5'-GACTGTGACGAGTTGGCTGA-3'	5'-CTGGAGAGGAGCAGAACTGG-3'
β-Actin	5'-GACTGTGACGAGTTGGCTGA-3'	5'-CTGGAGAGGAGCAGAACTGG-3'

Abbreviation. RANKL = receptor activator of nuclear factor kappa-B ligand; RUNX2=Runt-related transcription factor 2

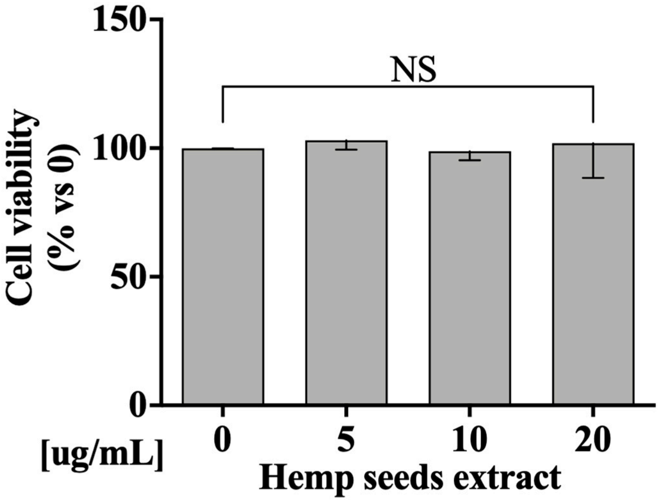

Figure S1. HSE does not increase cell viability of Saos-2 cells. Semi-confluent cultures of human osteoblast-like cells (Saos-2) incubated with HSE 5, 10, 20 ug/mL for 24 h. Cell viability determined by MTT assay. Data are represented as mean ± SD.

Abbreviations: MTT assay, 3-(4,5-dimethylthiazol-2-yl)-2,5-diphenyltetrazolium bromide assay.
